# Delayed Tooth Replantation after Traumatic Avulsion: A Case Report

**Published:** 2008-07-10

**Authors:** Zohreh Khalilak, Mahshid Shikholislami, Ladan Mohajeri

**Affiliations:** 1 *Department of Endodontics, Dental School, Iranian Center for Endodontic Research, Islamic Azad University, Tehran, Iran*; 2 *Department of Endodontics, Dental School, Islamic Azad University, Tehran, Iran*

**Keywords:** Calcium Hydroxide, Replantation, Resorption, Tooth Avulsion, Traumatic Injury

## Abstract

Avulsion is a serious injury which causes damage to dental and supportive tissues, ranging from 1-16 % among dental injuries and it mostly occurs in maxillary incisors. This report presents a case of replantation of a traumatically avulsed central incisor. The left central incisor of an 8 year-old boy with open apex was avulsed and was left in unclean and dry conditions. Tooth was replaced after 270 min and splinted. After 24 hours, tooth was treated endodontically. The calcium hydroxide paste was applied as intracanal medicament. After one year the calcium hydroxide was not replaced and was maintained in the canal, permanently. The tooth followed for 5 years. During follow up, the tooth kept stable. However, the resulted dent alveolar ankylosis prevented growth of the alveolar process. Spite of the fact that in children, replacement resorption leads to the loss of ankylosed teeth within 1-5 years; this tooth has remained in a stable, infra-position for 5 year and in functional position after coronal restoration. However, in such cases other treatments such as decoronation should be considered.

## INTRODUCTION

Traumatic injuries to newly erupted permanent anterior teeth are common during childhood and 0.5-16% of the 7-70 year-old group experience tooth avulsion (1). The most preferable management for the avulsed tooth is immediate replantation, within 20-30 min after injury or keeping in storage media until dental visit (2). Viability of the remaining periodontal ligament cell on the root surface of a replanted tooth is the most important factor in determining its prognosis (3). Some studies have found that extended extra-alveolar time is a good predictor of resorption (2-4), while others have not (4). All these studies, however, have shown that those teeth kept dry, eventually developed root resorption. Total extra-alveolar time has less effect on the outcome provided when the tooth has been stored in a wet medium (4). Andreason has reported that if the tooth has been out of the mouth for more than 2 hours, there is a 95% chance of external resorption (5). Nevertheless, replantation of an avulsed tooth for a child must be done even if the prognosis is not good.

The purpose of this case report was to present the clinical and radiographic condition of an avulsed and replanted maxillary central incisor that was treated with long term calcium hydroxide after an extended dry extra-alveolar period in a growing patient.

## CASE REPORT

A healthy 8 year-old boy was referred to the endodontic clinic for the treatment of traumatically avulsed maxillary left central incisor after a car accident. The tooth had been found 4 hours after the accident. The upper right central incisor tooth also suffered from luxation.

Thereafter, the tooth was replanted by a general practitioner after 30 min. The dentist rinsed the socket and tooth with saline and prophylactic antibiotic therapy had been prescribed for one week. The patient was examined clinically and radiographically. An intraoral examination showed no laceration, abrasions, and contusion of the soft tissues. Maxillary central incisors were wire splinted with acid etch composite resin attached to the adjacent teeth for one week.

**Figure 1:**
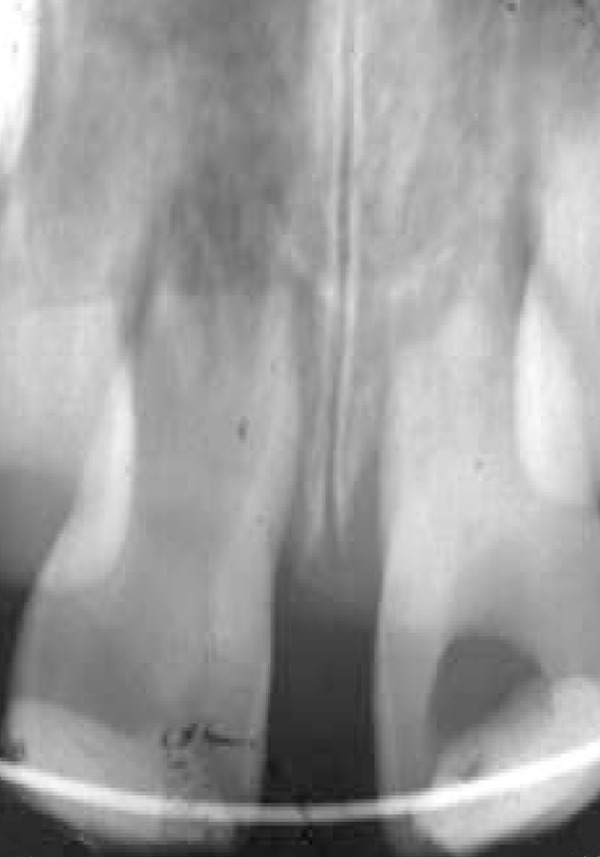
Tooth replanted and splinted after 4.5 h extraoral dry storage

**Figure 2:**
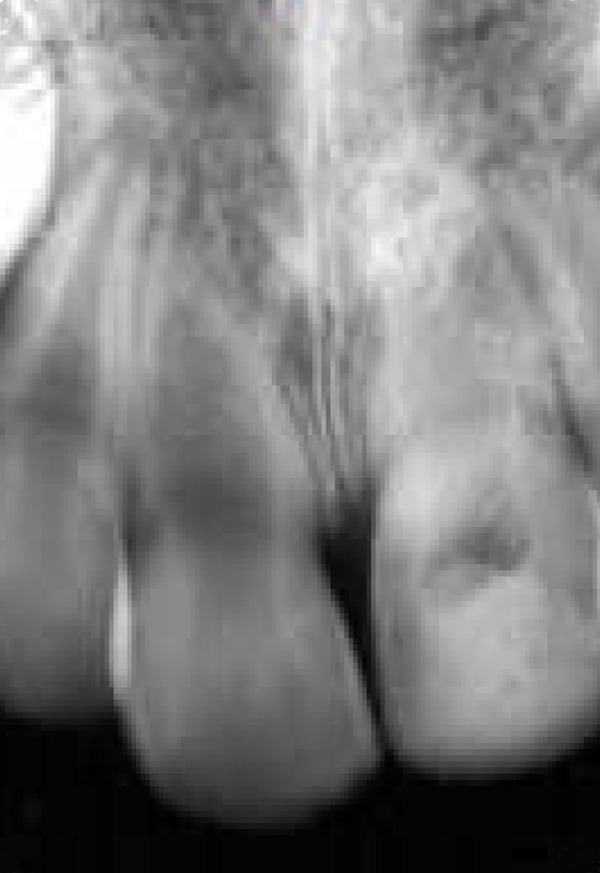
Radiograph taken one year after avulsion

**Figure 3:**
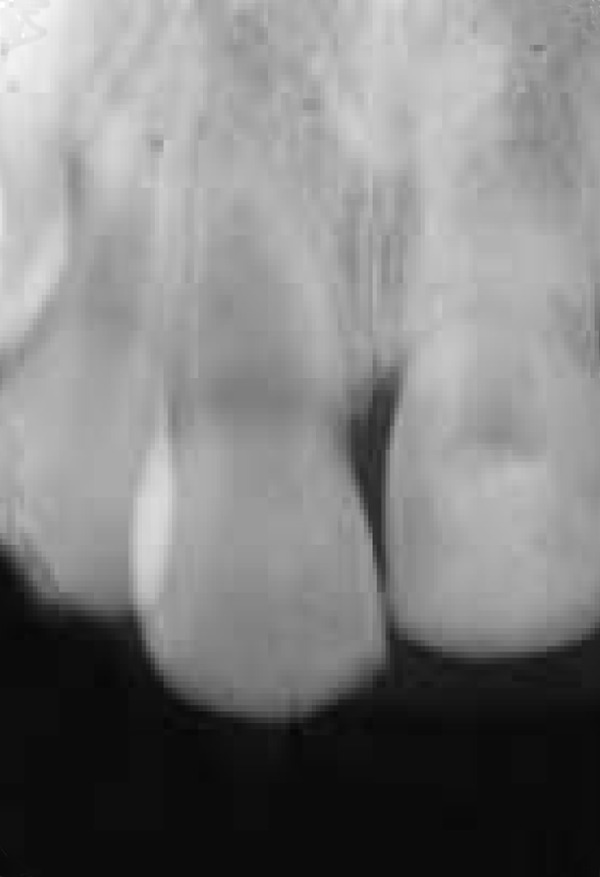
Follow-up radiograph taken 4 years after avulsion

The next day, the replanted tooth was endodontically treated. The canal was instrumented and irrigated with normal saline and 2% sodium hypochlorite solution, alternatively. Calcium hydroxide paste (Merk, Darmstadt, Germany) was placed in the root canal (Figure 1). In order to gain coronal seal, the tooth was filled with composite resin (Charisma, Kultzer, Germany). The calcium hydroxide paste was replaced three times during a year (3, 6, and 12 months). One year later the apexes of both central incisors were closed (Figure 2).

The maxillary central incisors were radiographi- cally and clinically examined during scheduled appointments after the first year (Figure 3). After one year the calcium hydroxide paste replaced for the last time and it was remained in the tooth. The tooth was restored with composite resin permanently. During the treatment, the vitality of right central incisor has been checked.

**Figure 4:**
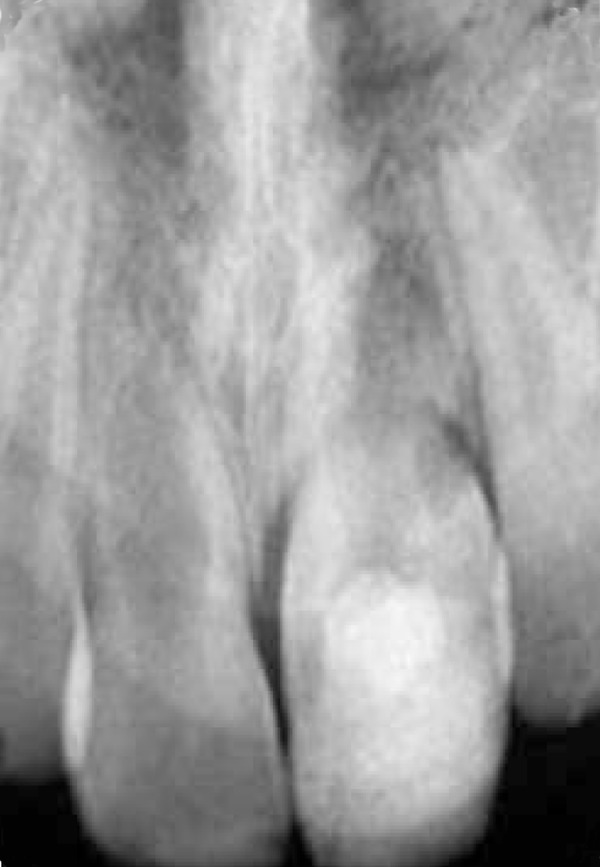
Radiograph taken 5 years after avulsion

After 5 years, the avulsed tooth kept in functional position. However, the avulsed tooth is infra- position because an ankylosis interferes with the vertical growth of the alveolar process. Although the space of pulp canal of the right central incisor is as wide as the first years, the growth has been completed; the apex is closed and the tooth remains vital (Figure 4).

## DISCUSSION

In the present case, the extra oral time was 270 min without placement in any medium. When a tooth has had an extra-oral dry time of greater than 60 min, the periodontal ligament is not expected to survive. Pre-treatment of such a tooth, prior to its replanting, will render it more resistant to resorption. If the tooth remained dry for more than 60 min with no consideration for preserving the periodontal ligament, the endodontic therapy could be performed extra orally (4,6). As a matter of fact, the patient was referred to endodontic clinic, and the tooth had been reimplanted and splinted. In the present case, with extra-oral dry time more than 4 hours and without any pre-treatment, the expected prognosis was poor. Donaldson and Kinirons (7) found that the risk of early resorption is increased in teeth that have additional damage or contamination of the root or are kept in dry conditions for longer than 15 min. They found that dry time is the most critical clinical factor associated with the development of post replantation root resorption. A previous study by Kinirons *et* *al. *(8) indicated that the risk of resorption increased dramatically after 5 min of dryness, with the probability of resorption increasing by 29% for every additional 10 min of dryness. According to the International Association of Dental Traumatology (IADT), replantation of avulsed permanent tooth with open apex with extra-oral time more than 60 min is not indicated (9). Trope (10) reported when severe additional damage cannot be avoided and osseous replacement of the root is considered certain, steps are taken to slow the replacement of the root by bone to maintain the tooth in the mouth for as long as possible. So, in this case the appropriate treatment was performed.

First of all, the root canal was cleaned thoroughly and then, the day after avulsion, calcium hydroxide has been placed in the canal in order to prevent inflammatory external root resorption. The clinical time recommended for the use of calcium hydroxide in avulsed teeth has been controversial.

**Figure 5:**
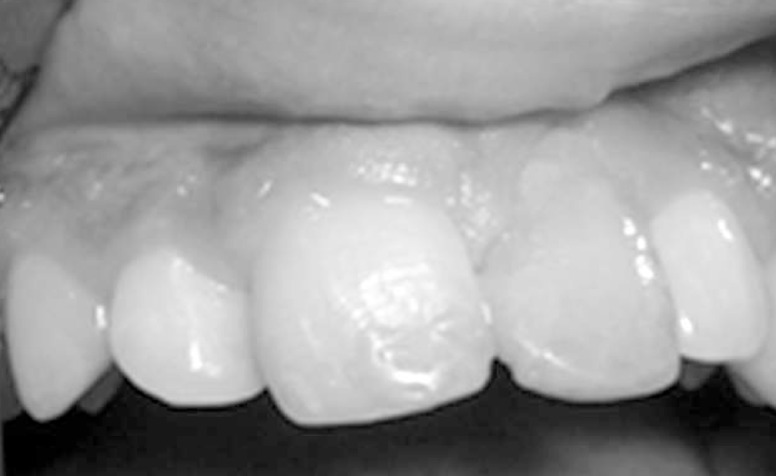
Clinical aspect after 5 years

Long-term clinical studies and case reports demonstrated that the use of calcium hydroxide for avulsed teeth resulted in an extremely high rate of success (2). On the other hand, a long- term calcium hydroxide treatment regimen may have some disadvantages. This treatment requires multiple appointments extending over a long period of time, making patient cooperation a critical factor. Without patient's cooperation, the root canal may ultimately become infected, resulting in probable tooth loss (2). During five years, there is no obvious external inflammatory root resorption but ankylosis was observed (Figure 5).

While appropriate endodontic therapy is effective in the treatment of external inflammatory resorption, replacement resorption cannot be arrested or repaired. Although there is no treatment known for dentoalveolar ankylosis, there are many different alternative treatment suggested in the literature such as keeping the ankylosed tooth or root, extraction and replacement by another tooth orthodontically, autotransplantation, implants or other prosthetic therapy (11). However, loss or extraction of teeth in a growing alveolar process will result in resorption of the crest and loss of development in that region. For this reason, extraction followed by prosthetic treatment in early ages should be avoided. Also, in young growing patients, fixed prostheses should be avoided possible because they may interfere with growth and development of tissues. When the tooth has been lost or extracted, it can be replaced by moving an adjacent incisor, usually a lateral incisor, into the space (11). It has been shown that cases with unilateral space closure more often resulted in a higher degree of patient dissatisfaction compare with patients where implant treatment was chosen (12).

Implants treatment is not in agreement with growth age because it will interfere with growth in the same way as an ankylosed tooth, resulting in infra-position. Diaz *et al. *has recently suggested the surgical technique of decoronation for the management of infra- positioned ankylosed replanted incisors in young patients (13). Decoronation is recommended for ankylosed teeth to preserve the contour of the alveolar ridge, and when the infra-position of the tooth crown is more than 1 mm (8). In this case calcium hydroxide paste is still in canal and it did not replaced after one year. Clinical and radiographical follow-ups are still being continued.

## CONCLUSION

Although replacement resorption leads to the loss of ankylosed teeth within 1-5 years, this tooth has remained in a stable, infra-position but functional situation for more than 5 years. So, it is wise to maintain calcium hydroxide in canal to save the tooth as long as possible. However, in such cases other treatments such as decoronation should be considered to preserve the normal size of the alveolar bone.
